# Cooking for Vitality: Pilot Study of an Innovative Culinary Nutrition Intervention for Cancer-Related Fatigue in Cancer Survivors

**DOI:** 10.3390/nu12092760

**Published:** 2020-09-10

**Authors:** Cheryl Pritlove, Geremy Capone, Helena Kita, Stephanie Gladman, Manjula Maganti, Jennifer M. Jones

**Affiliations:** 1 Applied Health Research Centre, Li Ka Shing Knowledge Institute, St. Michael’s Hospital, Toronto, ON M5G 1B1, Canada; Cheryl.Pritlove@unityhealth.to; 2 School of Kinesiology and Health Science, York University, Toronto, ON M3J 1P3, Canada; 3 Cancer Rehabilitation and Survivorship Program, Princess Margaret Cancer Centre, Toronto, ON M5G 2M9, Canada; Geremy.Capone@uhnresearch.ca (G.C.); Stephanie.Gladman@uhn.ca (S.G.); 4 MD Program, Faculty of Medicine, University of Toronto, Toronto, ON M5S 1A8, Canada; helena.kita@mail.utoronto.ca; 5 Department of Biostatistics, Princess Margaret Cancer Centre, Toronto, ON M5G 2M9, Canada; Manjula.Maganti@uhn.ca; 6 Department of Psychiatry, Faculty of Medicine, University of Toronto, Toronto, ON M5T 1R8, Canada

**Keywords:** cancer survivorship, cancer-related fatigue, nutrition, culinary

## Abstract

(1) Background: Cancer-related fatigue (CRF) is one of the most prevalent and distressing side effects experienced by patients with cancer during and after treatment, and this negatively impacts all aspects of quality of life. An increasing body of evidence supports the role of poor nutritional status in the etiology of CRF and of specific diets in mitigating CRF. We designed a group-based two session culinary nutrition intervention for CRF, Cooking for Vitality (C4V), aimed at increasing understanding of how food choices can impact energy levels and establishing basic food preparation and cooking skills as well as the application of culinary techniques that minimize the effort/energy required to prepare meals. The purpose of this pilot mixed-method study was to evaluate: Feasibility of the experimental methods and intervention; acceptability and perceived helpfulness of intervention; and to obtain a preliminary estimate of the effectiveness of the intervention on fatigue (primary outcome), energy, overall disability, and confidence to manage fatigue (secondary outcomes). (2) Methods: Prospective, single arm, embedded mixed-methods feasibility study of cancer survivors with cancer-related fatigue was conducted. Participants completed measures at baseline (T0), immediately following the intervention (T1), and three months after the last session (T2). Qualitative interviews were conducted at T2. (3) Results: Recruitment (70%) and retention (72%) rates along with qualitative findings support the feasibility of the C4V intervention for cancer survivors living with CRF (program length and frequency, ease of implementation, and program flexibility). Acceptability was also high and participants provided useful feedback for program improvements. Fatigue (FACT-F) scores significantly improved from T0–T1 and T0–T2 (*p* < 0.001). There was also a significant decrease in disability scores (WHO-DAS 2.0) from T0–T2 (*p* = 0.006) and an increase in POMS-Vigor (Profile of Mood States) from T0–T1 (*p* = 0.018) and T0–T2 (*p* = 0.013). Confidence in managing fatigue improved significantly from T0–T1 and T0–T2 (*p* < 0.001). (4) Conclusions: The results suggest that the C4V program was acceptable and helpful to patients and may be effective in improving fatigue levels and self-management skills. A randomized controlled trial is required to confirm these findings.

## 1. Introduction

Advances in biomedical treatment are changing the nature of cancer as a disease, with individuals increasingly surviving the acute phases of illness [1]. The prevailing chronicity of cancer means that patients are living longer with the consequences of their illness, including the physical and psychosocial side-effects of its treatment [2,3]. Cancer-related fatigue (CRF) is defined as “… a distressing persistent, subjective sense of physical, emotional and/or cognitive tiredness related to cancer or cancer treatment that is not proportional to recent activity and interferes with usual functioning” [4]. It is one of the most prevalent and distressing side effects experienced by patients with cancer during and after treatment. During treatment, acute CRF is experienced by the vast majority of patients and can negatively impact functioning in daily, social life, family care, mood, sleep, cognitive and physical function, sense of self, and quality of life (QoL) [5,6]. Following the completion of cancer treatments, CRF persists in up to 40% of disease-free cancer survivors and this impacts work productivity and adoption of healthy lifestyle behaviors further adding to disability [5,6,7,8,9,10]. 

In response to the increasing burden of CRF, there has been a surge in research focused on identifying contributors to CRF and designing interventions targeting these etiological factors. An increasing body of evidence supports the role of poor nutritional status in the etiology of CRF and the role of specific diets (i.e., targeting nutritional deficiencies and metabolic disturbances) in mitigating CRF. For example, well-documented nutritional consequences of cancer and its treatment have been linked to CRF including altered metabolism and chronic inflammation, anorexia, unintentional weight loss, loss of lean muscle mass, nutritional deficiencies, and increased adiposity or obesity [11,12,13,14,15,16,17,18,19,20,21]. Dietary intake that is high in anti-inflammatory foods including fruits, vegetables, whole grains, lean proteins, spices, and healthy fats with an emphasis on omega-3 fatty acids and low in saturated fat is recommended for cancer survivors [22,23,24] and is also associated with lower levels of CRF [25,26,27,28,29,30,31]. In a recent Phase II clinical trial, Zick and colleagues [32] found that participants randomized to a three-month diet rich in fruit, vegetables, whole grains, and omega-3 fatty acid-rich foods had significantly improved fatigue and sleep compared to an attention control. These findings suggest that modifications to dietary intake may be a potential target for intervention and may attenuate CRF through these mechanisms [25,26,29]. Unfortunately, only a small minority of cancer survivors are meeting the nutrition guidelines for fruits and vegetables [33,34] or consuming a high quality diet [35]. Furthermore, while patients report a high interest in and need for nutritional information, many do not have access to high quality nutrition information or counselling during or after cancer therapy [36]. 

Despite links between nutrition and CRF, and multiple guidelines suggesting nutritional consultation as a possible treatment option for CRF [37,38,39,40], there are no evidence-based guidelines for the nutritional management of CRF. In addition, there are a limited number of nutrition-focused fatigue self-management programs that have been developed and tested [11,41]. To date nutritional interventions for the management of CRF have primarily focused on face-to-face or telephone-based nutritional counselling from a registered dietitian [41]. Although nutritional counselling may be an effective method of providing individualized support [42], access to registered dietitians who have expertise in oncology may be limited [42], particularly within the context of a growing population of cancer survivors. In addition, the provision of information alone has been found to be ineffective to bring about sustained behavior change [43]. Furthermore, patients managing chronic conditions are often unsatisfied with dietetic advice on “healthy eating” because “it didn’t make sense in my daily life” [44] and many lack the confidence and ability to prepare and cook healthy foods [45]. 

Group-based culinary nutrition interventions that teach food preparation and practical cooking skills along with nutrition information have been shown to improve participant’s confidence in cooking and diet quality [46,47,48,49,50,51,52]. Furthermore, group-based interventions provide the opportunity for interaction between members, the provision of social support [53,54,55,56], and social validation and modelling [57,58]. A culinary nutrition intervention for CRF is an innovative approach that could offer nutritional counselling related to CRF and the opportunity to build cooking skills and confidence. Given that fatigue often limits cancer survivor’s ability to implement existing CRF management recommendations [59], energy conservation skills (a recommended approach to managing CRF) [60] could also be embedded through teaching recipes that minimize exertion, preparation, and clean up. 

Despite the benefits of cooking classes as a medium for a nutritional intervention and evidence supporting a link between CRF and nutrition, to date no culinary nutrition intervention for CRF has been developed or evaluated. To address this gap, we designed a group-based intervention, Cooking for Vitality (C4V), aimed at helping cancer survivors self-manage and reduce their CRF. The purpose of this mixed-method pilot study was to evaluate the feasibility, acceptability, and preliminary impact of C4V.

## 2. Methods

We conducted a prospective, single arm, embedded mixed-methods feasibility study of cancer survivors with cancer-related fatigue at the Princess Margaret Cancer Centre in Toronto, Canada. Participants completed measures at baseline (T0), immediately following the intervention (T1), and 3 months after the last session (T2). Qualitative interviews were conducted at T2. The research aims were to assess the: (1) Feasibility of the experimental methods and intervention; (2) acceptability and perceived helpfulness of intervention; and (3) to obtain a preliminary estimate of the effectiveness of the intervention on fatigue (primary outcome), energy, overall disability, and confidence to manage fatigue (secondary outcomes). 

This study was approved by the University Health Network Research Ethics Board (REF#16-5697) and all participants provided written informed consent.

### 2.1. Intervention

**Figure 1:**
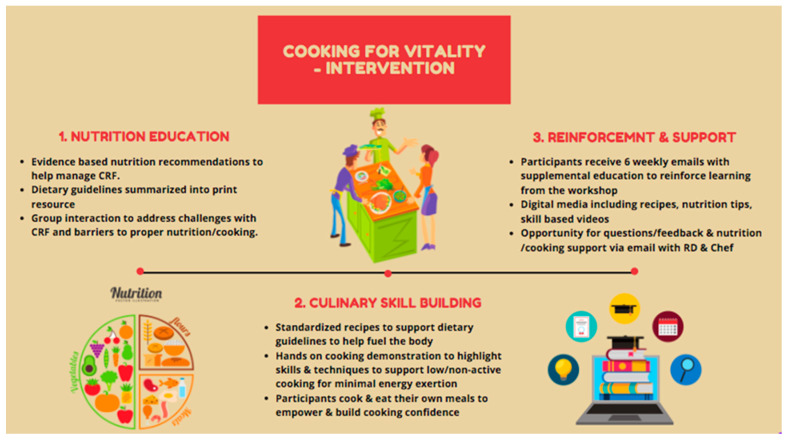
Description of C4V intervention. CRF: Cancer-related fatigue.

Cooking for Vitality (C4V) is a culinary nutrition intervention that was developed by the Princess Margaret Cancer Rehabilitation and Survivorship Program team, including dietitians, cancer rehabilitation clinicians, and researchers with expertise in the area of Cancer-related fatigue (CRF), as well as the wellness chef. Culinary students from George Brown College also aided in the development of recipe content. The intervention was delivered in a kitchen teaching lab within the Princess Margaret ELLICSR Centre for Health, Wellness and Cancer Survivorship. The development of C4V was informed by several theoretical frameworks including Social Cognitive Theory (SCT), Social Learning Theory (SLT) [61], and Experiential Learning Theory [62]. In addition, the intervention draws upon several established behavior change techniques that have been associated with long-term positive outcomes for cooking skills and diet [63] including providing information on consequences of behavior in general; providing instruction on how to perform the behavior and where/when to carry out the task; demonstration of the skills; identification of barriers/problem solving; and prompting practice [64]. Learning objectives for intervention participants included: (1) An enhanced understanding of how food choices can impact energy levels and (2) establishing basic food preparation and cooking skills as well as the application of culinary techniques that minimize effort/energy required to prepare meals (see Figure 1).

C4V intervention is group based (maximum of 10 participants) and consists of two structured hands-on cooking classes that are delivered at ELLICSR by the Cancer Rehabilitation and Survivorship program’s wellness chef and registered dietitian (RD). Each class was 1.5 h in length and included a mix of didactic teaching, demonstrations, and hands-on participation. The first class provided an overview of CRF and nutritional counselling regarding CRF. Recommendations were based on the fatigue reducing diet developed by Zick and colleagues [31] and also stressed the importance of hydration, protein, anti-inflammatory foods, and a balanced plate [24,29,65]. Participants were then guided though an interactive cooking class and prepared three recipes that have been tested and developed to address CRF. Participants learned about the nutritional benefits and recommendations of each recipe and the ingredients during the class. Participants were also provided with a recipe package complete with ingredients list, directions, and nutrition tips to help them follow along during the class and reinforce the knowledge afterwards. Participants were encouraged to ask questions and to think about potential barriers and discuss potential solutions and developed goals for themselves. Following the first class, participants received weekly (x6) supporting emails. The emails reinforced key information provided during the first class and provided a new recipe for them to try each week. The second class was held 6 weeks later and began with a review of the main learning points followed by a group discussion around any experiences the participants had trying the recipes. This was followed by an interactive cooking class with 3 additional recipes. Participants were allowed to be accompanied by their spouse/partner/caregiver to observe the cooking demonstrations if desired. 

### 2.2. Participants and Procedure

Participants were adult cancer survivors who had completed their cancer treatment and were experiencing cancer-related fatigue. Recruitment occurred through study posters at the hospital and through social media (Twitter). Interested participants contacted the study coordinator (phone or email) and were provided with information about the study and intervention and screened for eligibility. Eligibility criteria included: Age > 18 years; completed primary cancer treatments (adjuvant hormone therapy was permitted); experiencing self-reported cancer-related fatigue symptoms (no pre-defined cut-off score); ability to understand and read English; access phone/computer to view intervention emails and videos; and access to a kitchen and willingness to cook. Participants were excluded if they had significant food allergies, were unwilling to attend the in-person classes, or not willing to provide consent. 

Eligible participants that provided verbal consent were scheduled for their first class. One week prior to the first class, the consent form was mailed to participants for review and to complete and return. Upon receiving signed consent, participants had the option to complete the baseline questionnaire (T0) either online using a Canadian-based secure Internet site (www.fluidsurveys.com) or in-person prior to the start of the first class. Basic demographic information was collected on the baseline questionnaire and a chart review was conducted to collect data on cancer diagnosis and treatment history. Participants also completed the questionnaire package at the second class (T1) and 3 months after the second class (T2).

### 2.3. Study Outcomes

#### 2.3.1. Demographic and Clinical Data

Basic demographic and clinical information were obtained through self-report and chart review at baseline.

#### 2.3.2. Primary Outcome: Feasibility and Acceptability

Feasibility of the study intervention and study procedures were assessed by tracking (1) *recruitment and retention rates*. Reasons for participant withdrawal were also collected, (2) *intervention adherence* was captured through class attendance, and (3) *capture of outcomes* including the number of participants who completed the clinical outcome (primary and exploratory) assessments and documented rates of missing data. A priori, we determined that feasibility would be confirmed with: (i) >70% intervention adherence and (ii) attrition rate <30%. (4) Feasibility was also informed by participants’ feedback regarding the adoption and implementation of the intervention during qualitative interviews.

To assess acceptability and inform future program refinement, we conducted semi-structured, qualitative telephone interviews with participants following intervention completion. The interview guide was informed by the overarching aims of the study and designed to align with questionnaires administered. All interviews were conducted by a trained qualitative methodologist (CP). A total of 21 individuals who completed the C4V intervention completed a qualitative interview 3 months post completion, at which point, thematic saturation [66] was reached. Individual interviews lasted between 30–65 min in length. 

#### 2.3.3. Secondary Outcomes: Exploratory Clinical Outcomes 

Standardized questionnaires were used to obtain a preliminary estimate of the impact of the Cooking for Vitality intervention on fatigue (primary), energy, overall disability, and confidence to manage fatigue (secondary). Questionnaires were completed by participants at baseline (T0), at the end of the intervention (T1), and 3 month after the intervention (T2). 

(1) *Fatigue* was measured using the 13-item Functional Assessment of Chronic Illness Therapy—fatigue (FACIT—fatigue) scale [67,68]. Items are rated on a 5-point Likert scale (0–4) resulting in a total possible score of 0–52 with higher scores indicating less fatigue. A cut-off of <34 was used to indicated significant CRF [69]. 

(2) *Energy level* was measured using the 6-item Profile of Mood States—Vigor subscale (POMS—vigor) [70,71] (ref). Respondents indicate the degree to which each adjective describes their mood during the previous week using a 5-point Likert scale (0–4) (score range 0–24). 

(3) *Disability* was measured using the 12-item World Health Organization Disability Assessment Schedule 2.0 (WHO-DAS 2.0) [72,73]. Respondents rate their difficulty in engaging in particular activities on a scale from “none” (no difficulty) to “extreme or cannot do” in 6 domains of functioning. Scores range from 12 to 60, where higher scores indicate higher disability or loss of function. 

(4) *Confidence in managing fatigue* was measured using a 5-item study constructed questions adapted from previous confidence tools [47,74,75]. Patients were asked to rate how confident they felt managing fatigue on an 11-point numeric rating scale ranging from 0 (not at all confident) to 10 (very confident). Questions included: I can manage my fatigue; I know which foods worsen fatigue; I know which foods will improve my energy levels; I am able to eat a variety of foods; I can prepare foods in ways that help me from being too tired. Total confidence scores range from 0–50. 

(5) Qualitative interviews garnered further insight into the impact of the C4V intervention on fatigue and fatigue management.

### 2.4. Data Analyses

The target sample size for this pilot study (*n* = 40) was based on our primary aim to assess the feasibility of the methods and acceptability of the C4V intervention and is consistent with sample size recommendations for pilot studies [76]. Participant demographics and clinical characteristics were analyzed using descriptive statistics. 

Numbers and proportions of those who contacted the study coordinator and were included/excluded and dropout and attrition rates were calculated. Adherence to the intervention was expressed as a percentage who attended one (partially adherent) or both (adherent) of the classes. The percentage of patients with complete capture of all outcomes (i.e., no missing data) were calculated. Feasibility was also measured by analyzing participants’ adherence to and implementation of the intervention (see approach to qualitative analysis below).

Statistical analyses were performed on data from the 58 participants who completed baseline measures. For all exploratory clinical outcomes that were continuous in nature, estimated means at each time point were calculated and compared using linear mixed effects models. The proportion of participants scoring below fatigue cut-off (<34), indicating significant CRF, was also examined across time points using GEE (Generalized Estimating Equations) procedures and the corresponding odds ratios were reported. All statistical analyses were conducted using SAS software, version 9.3 (SAS Institute, Cary NC, USA). Statistical significance is considered as *p* < 0.05.

Qualitative interviews were digitally recorded and transcribed verbatim. Transcripts were coded line by line to explore emergent themes and derive analytic concepts. Two researchers (C.P. and H.K.) initially independently coded the data, and then held meetings to develop, refine, and reach consensus on key codes and themes. Preliminary themes were identified as those that related to the study objectives, were discussed consistently within an individual interview, as well as those that were discussed repeatedly between participant interviews. Following this process, the transcripts were critically analyzed using the constant comparison method [77] to better understand key differences and similarities within and between participants. This process allowed us to develop and refine the codebook, which was used for a third round of coding using NVivo data management software, version 11 (QSR International, Melbourne, Australia). This was used to support data management and produce thematic reports of interview quotations.

## 3. Results

**Figure 2:**
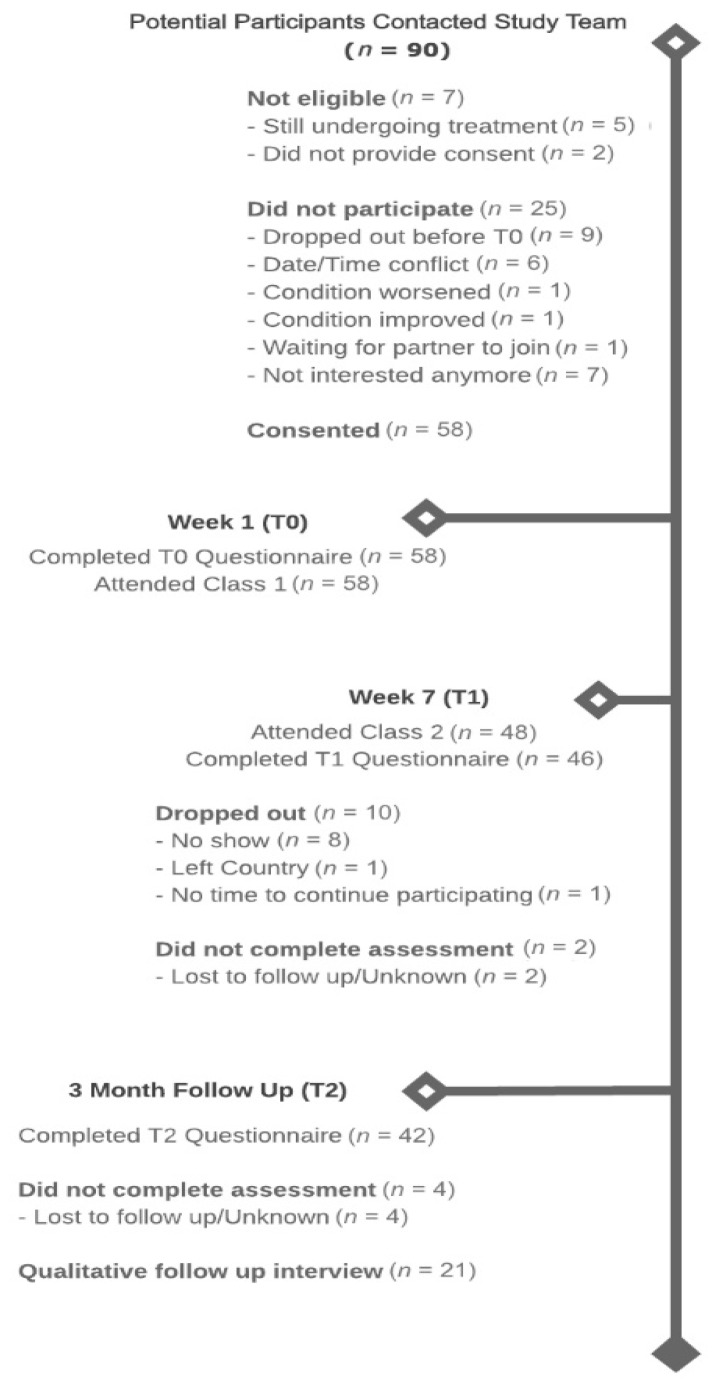
Study Flow Chart.

The study flow diagram is presented in Figure 2 and participant demographic and clinical characteristics are in presented in Table 1. 

### 3.1. Feasibility

Recruitment occurred between November 2016 and August 2019, during which time 90 potential participants contacted the study coordinator. Of these, seven were deemed ineligible (8%), 16 (18%) declined participation, and nine (10%) expressed interest but then dropped out before completing the baseline assessment. Reasons for ineligibility included still undergoing primary cancer treatment (*n* = 5) or not willing to provide consent (*n* = 2). The reasons for declining participation were date/time conflicts (*n* = 15), not interested (*n* = 7), change in condition (*n* = 2), and availability of partner (*n* = 1). A total of 58 (70%) participants completed the baseline assessment (T0) and attended the first class and 48 (83%) participants attended class two. A total of 46 (79%) participants completed the post-intervention assessment (T1) and 42 (72%) completed the three-month post-intervention assessment (T2).

The qualitative findings support the feasibility of the C4V intervention for cancer survivors living with CRF. Three key themes emerged from participants’ qualitative interviews pertaining to feasibility, these included program length and frequency, ease of implementation, and program flexibility. A summary of these themes is presented below. A more fulsome analytic description of each of the themes along with illustrative quotes are summarized in Table 2.

Most participants felt the program length and frequency of in-class sessions were reasonable. Although, some explained that additional in-class sessions would have been helpful to further indoctrinate culinary practices. This was particularly true for those who entered the program with less nutritional knowledge and culinary skills. Participants with and without previous nutritional knowledge and culinary expertise explained that the nutritional information and culinary strategies learned through the program were easy to understand and apply within the home. Most participants continued to make recipes and employ culinary skills for the duration of the intervention and beyond (as noted in the three months follow-up interview). Participants who had timing conflicts as well as those who were feeling too unwell or fatigued to attend their scheduled in-class session appreciated the opportunity to join other C4V groups. 

### 3.2. Acceptability

The impacts of fatigue on the everyday lives of the participants in this study were diverse and far reaching, affecting their physical and emotional health as well as their social and vocational lives. Participants felt strongly about the association between food, health, and illness-management, yet many experienced difficulty navigating nutritional information on food and cancer and applying this to their fatigue-specific needs and limitations. With limited options available, many participants described relying on convenience or fast food out of necessity, despite reservations about the consequences for their health. On the heels of these conversations, most emphasized the need for greater professional care and guidance to support them in their fatigue management, for which diet was believed to play a significant role. The need for this support provided the catalyst to join the C4V program.

Two key themes emerged from participants’ qualitative interviews pertaining to acceptability, these included satisfaction with the C4V program and areas for program improvements. A summary of these themes is presented below. A more fulsome analytic description of each of their sub-themes along with illustrative quotes are summarized in Table 3.

### 3.3. Satisfaction with the C4V Program

All of the participants demonstrated a high degree of satisfaction with the C4V program, describing the various ways in which they found the program to be of value. This included access to expert information and personalized support from a chef and registered dietitian; the provision of tips, tricks, and tools to facilitate cooking while experiencing fatigue; experiential learning; and social support. 

Participants explained that nutritional information in the context of cancer was widely accessible, however, it was also diverse, non-specific (e.g., rarely side-effect specific), and often contradictory. Navigating this sea of information and being able to adequately assess its credibility was challenging and ultimately hampered confidence in making decisions around dietary practices. Participants appreciated that the C4V program provided access to trained professionals who could provide credible, reliable, and personalized nutritional and culinary knowledge, alleviating some of this confusion, and paving a clearer path forward for behavior change. 

Participants also explained that standing for long periods of time, making multiple meals a day, and cleaning up following meal preparation no longer seemed feasible in light of the limitations posed by their CRF and many participants turned to pre-prepared or fast food meals as a result. The C4V program provided participants with culinary strategies (e.g., batch cooking/freezing, parchment paper/one pot meals, pre-cut, washed, and frozen fruits and vegetables) to help overcome common barriers to cooking for those living with CRF, and ultimately enhanced their capacity for meal preparation. 

Finally, the opportunity to apply nutritional information and culinary strategies in a hands-on, class-based setting was crucial to participants’ capacity to retain nutritional information as well as practice and refine newly-acquired culinary techniques and skills. Access to this kind of experiential learning helped participants to more easily transition these skills from the classroom to the home. The group-based environment also permitted cancer survivors to interact with as well as learn from each other, enhancing the overall educational experience as well as normalizing and validating their experiences with CRF, which some explained was challenging given the invisibility of this side effect. 

### 3.4. Areas for Program Improvement

The participants in this study found the C4V program to be of value, with all of the participants explaining that they would recommend this program to other cancer survivors. However, recognizing the infancy of the program, some also suggested ways in which the program could be improved. Specifically, participants explained that the program could benefit from more one-on-one consultation, a graduated or multi-tiered approach to program delivery, more in-person cooking sessions, and a varied approach to the provision of support materials. 

Given the unique needs and skill levels of each individual, some participants explained that initial one-on-one consults could help to further personalize the content of group sessions. To further personalize the program, some suggested that a graduated, multi-tiered program (e.g., beginner, intermediate, advanced) would allow participants to enter the program at a level they felt most comfortable with, while also permitting individuals to build upon and advance their knowledge and skills by graduating to different levels. Additional in-person cooking sessions were also suggested to help further indoctrinate nutritional information and culinary skills. And lastly, particularly for those who struggled with technology, it was explained that offering print materials and other mediums for information delivery (sending content via email alone), would be helpful. 

### 3.5. Exploratory Clinical Outcomes

**Figure 3:**
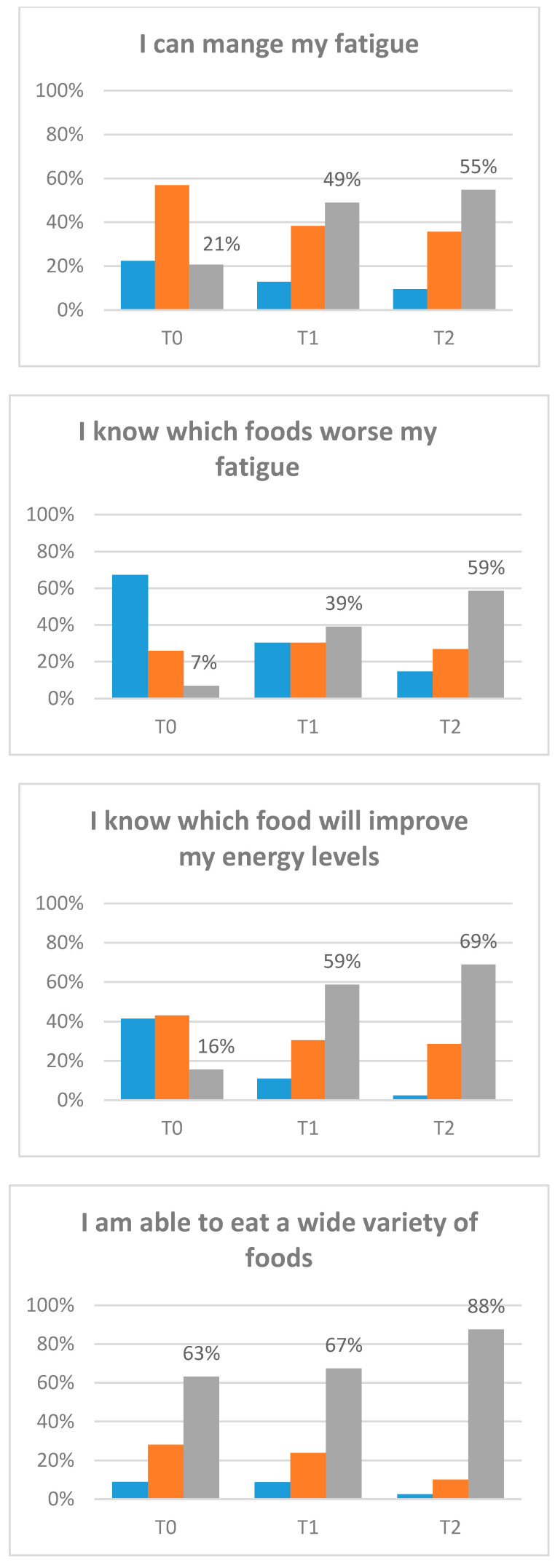

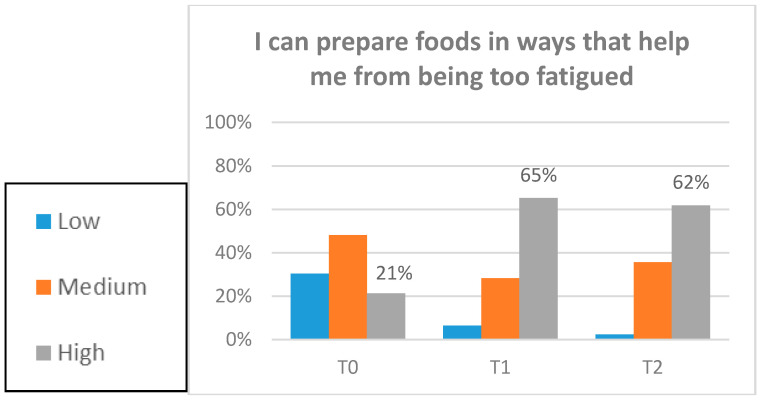
Confidence items. Participants completed the baseline assessment (T0). Participants completed the post-intervention assessment (T1). Participants completed the three-month post-intervention assessment (T2).

Preliminary estimates of the treatment effects for participants on fatigue, disability, energy, and confidence were evaluated (Table 4). Fatigue scores significantly improved from T0–T1 and T0–T2 (*p* < 0.001). This improvement was at a clinically important level [78]. Furthermore, the proportion of participants scoring at or below the clinical cut-off (<34), indicating significant fatigue, significantly decreased from 82.14% at T0 to 57.14% at T2 (*p* = 0.007). There was also a significant decrease in disability scores from T0–T2 (*p* = 0.006) and an increase in energy from T0–T1 (*p* = 0.018) and T0–T2 (*p* = 0.013). Confidence in managing fatigue improved significantly from T0–T1 and T0–T2 (*p* < 0.001). Change of confidence (categorical) by item is displayed in Figure 3. 

Qualitative findings supported the quantitative results, reinforcing the positive impact of an evidence-based culinary nutrition intervention on cancer survivors’ capacity to self-manage and reduce CRF. Four themes emerged from the qualitative interviews pertaining to program utility and impact, these included improvements in motivation, improvements in self-efficacy, and enhanced feelings of control, as well as overall improvements in fatigue and fatigue management. A summary of these themes is presented below. A more fulsome analytic description of these themes along with illustrative quotes are summarized in Table 5.

Recipes and culinary strategies provided through the C4V program made meal preparation feel more attainable, enhancing feelings of motivation and self-efficacy. This helped many of the participants to push through their fatigue to make “healthier choices” that they felt more confident with. Establishing a sense of control over ones diet was one way participants began to return to normal and gain an improved sense of control over their lives.

While some participants felt that the nutritional information and culinary skills acquired through the program and the dietary changes they made as a result had a direct impact on reducing their CRF, most described a more indirect pathway. Emphasizing the multifactorial nature of their fatigue (e.g., cancer itself, cancer treatments, anxiety and depression, reduced physical activity, changes in diet, and co-morbid conditions), most explained that energy conservation strategies and dietary changes improved overall energy levels, facilitating engagement in other activities (e.g., physical activity). The culmination of these changes improved mood and facilitated improved illness management, all of which were believed to contribute to improvements in CRF. 

## 4. Discussion

The current pilot study reports the findings for a group-based culinary nutrition intervention to help cancer survivors self-manage and reduce their CRF. Evidence from this study suggest that the C4V intervention is a feasible approach that is highly acceptable to cancer survivors living with CRF. The program garnered especially high ratings on measures of satisfaction and usefulness, with study participants rating their motivation and confidence to manage fatigue significantly higher upon program completion. Results from the intervention also show statistically and clinically significant improvements in fatigue management and overall experiences of CRF. Positive outcomes at three months, including improved confidence in culinary practices and fatigue management, as well as improvements in energy and overall feelings of fatigue, are encouraging indicators of the effectiveness of the C4V intervention.

Patients managing chronic conditions are often unsatisfied with nutritional advice due to a lack of fit with their everyday lives [44] and many lack the confidence and capacity to prepare and cook healthy meals [45,46]. Moreover, cancer survivors highlight that their fatigue often limits their ability to implement existing CRF management recommendations [59]. These findings are consistent with those of the current study which found that even among those with nutritional knowledge and culinary experience at baseline, the unique needs and limitations posed by cancer and CRF rendered previous approaches to meal preparation infeasible or unpractical. Participants emphasized their need for reliable nutritional information paired with culinary strategies, designed to address the limitations posed by CRF. The findings from this study point to a range of social and pedagogical factors that might account for the feasibility, acceptability, and positive impact of this evidence-based culinary nutrition program. 

In-person and remote (e.g., email and telephone) access to program instructors along with the availability of program-specific online resources for use in the home were among the key factors that supported participants’ adherence to and successful implementation of the intervention. Experiential and group based learning have been shown to support and motivate participants to change health-related behaviors [79], this was reinforced by the experiences of the participants in the current study. Specifically, experiential learning improved participants’ familiarity with specific foods and cooking techniques. Moreover, the nutritional and culinary education content focused on the provision of tips geared specifically to the needs and limitations of those living with CRF, removing barriers to healthy eating and home cooking. The role of the instructors was instrumental in building motivation, confidence, and competence in culinary practices, ultimately facilitating greater participation in meal preparation and enhancing consumption of foods that meet the nutritional needs of cancer survivors with fatigue [80,81]. The findings from this pilot study thus provide new insight into the value of adding a cooking component to nutritional education for cancer survivors living with CRF, and ultimately supports the benefits of cooking classes as a medium for a nutritional intervention [82,83,84,85].

## 5. Strengths and Limitations

This study provides useful information on the feasibility, acceptability, and impact of the culinary nutrition intervention for CRF thus addressing an existing gap in the literature. Although the results of this intervention are encouraging, there are several limitations that warrant consideration. While the sample size was adequate for a pilot study in which the primary outcome is acceptability and feasibility, the measure of impact should be considered preliminary and treated with caution. Furthermore, without a control group, other explanations for improved fatigue (e.g., elapsed time since diagnosis and treatment, participation in other rehabilitative programs such as exercise programs, etc.) must be considered. Encouragingly, the qualitative results supported the quantitative findings. A randomized controlled trial could provide further insight into the direct impact of the program. Secondly, while group-based sessions enhanced the overall acceptability of the intervention and are likely to reduce cost [83,86], a cost-effectiveness analysis was not done and may be needed to further measure program feasibility and scalability. While both male and female cancer survivors experience CRF [87], significantly more women than men participated in the C4V intervention. Given the gendered nature of domestic and culinary practices [88], the needs of men and women and the potential barriers they face to implementing nutritional knowledge and meal preparation may be different. Purposive recruitment strategies [89] should be implemented to attract more men to participate in culinary nutrition interventions [90]. Lastly, while results from the qualitative study demonstrate a high degree of acceptability, interviews were conducted with participants who remained in the study up until the three-month follow up point, demonstrating ongoing engagement. The perspectives of these individuals may not reflect the perspectives of those who did not complete the intervention. 

## 6. Conclusions

CRF can lead to diminished quality of life among cancer survivors. While preliminary, the results from the C4V intervention showed statistically and clinically significant improvements in fatigue management and the overall experiences of CRF. High retention and positive outcomes at three months are encouraging indications of the potential success of the C4V intervention. A randomized controlled trial with additional assessment of cost-effectiveness is warranted.

## Figures and Tables

**Table 1:** Participant characteristics.

**Age (Years)** mean (SD), range	58 (± 12.3), 25–86
**Sex *n* (%)**	
Male	7 (12%)
Female	51 (88%)
**Marital Status***n* (%)	
Married/Common Law	23 (40%)
Divorced/Separated	12 (21%)
Widowed	1 (2%)
Single	21 (37%)
**Cancer Site***n* (%)	
Breast	35 (61%)
Gynecological	7 (12%)
Gastrointestinal	2 (3.5%)
Genitourinary	3 (5%)
Endocrinology	3 (5%)
Hematology	4 (7%)
Head and Neck	1 (2%)
Central Nervous System	1 (2%)
Skin	1 (2%)
**Time Since Diagnosis (months)**	
mean (SD), range	24 (± 24.4), 0–176
**Cancer Treatments***n* (%)	
Surgery	46 (81%)
Chemotherapy	33 (58%)
Radiation Therapy	40 (70%)

SD: Standard deviation.

**Table 2:** Qualitative table for feasibility.

Themes	Analytic Note	Example Quote
Program length and frequency	Most participants felt that the program length and frequency of in-class sessions were appropriate, particularly in light of restrictions on energy posed by cancer-related fatigue (CRF). Although, some explained that they would have liked more in-class sessions, they appreciated that this could pose a barrier to those with more severe CRF.	“This (program length and frequency) was perfect for what we were trying to do and I think especially for the people who really were quite overwhelmed by their sickened stances I think they found it fun and positive and not purely demanding, which is important.” “I don’t think I have any suggestions (for program improvements). The program takes into consideration our fatigue and so it’s a good balance of learning, but not overdoing it in terms of time (commitment). I would have liked more in-person classes, but know that this could deter some people whose fatigue is really bad (from taking part in the program). So, I think it (Cooking for Vitality) is very balanced.”
Ease of implementation	All participants explained that recipes and culinary strategies acquired through the program were easy to understand and most described ease of implementing this knowledge and skill at home. Participants found that the positive environment in which recipes were acquired provided a “halo” effect to the content that further helped to motivate participants to implement during and beyond intervention.	“So I’m sure that, well I can only speak for myself, but I went away feeling “gosh, we made some interesting recipes there, I can do that!” and I will do that, because it’s, and probably the recipes acquired a halo from the context in which I first saw them.” “There is a mental barrier for people who don’t cook. We don’t like cooking, just to do it. But I attended that session, and it came together so easily, and it tasted so good. So what are you waiting for, just do it! So I started making baked fish with vegetables [at home] just like the way he [wellness chef] taught us. It was so simple and so healthy too.”
Program flexibility	Participants appreciated flexibility to attend other in-class sessions when timing conflicts and or restrictions posed by fatigue prevented them from attending their scheduled group sessions. Without this flexibility, some participants explained that they would not have been able to fully adhere to the intervention by attending both in-class sessions.	“One of the classes I had to miss because I just didn’t have the energy that day. Well, Geremy reached out to see how I was doing and let me know that if I wanted, and I did, I could sit in with another group so that I didn’t miss out on anything, so that was helpful because, well at least my fatigue isn’t predictable, so that flexibility is really important for program like this.”

**Table 3:** Qualitative table for accessibility.

**Theme: Satisfaction with the C4V Program**		
**Sub-Themes:**	**Analytic Note**	**Example Quote**
Expert information and personalized support from a chef and dietitian	All of the participants reinforced the value of having a central and credible source of information specific to the needs and limitations of those living with CRF. The delivery of this information from professionals with specific expertise in the area of food/nutrition and cancer was particularly important to reinforcing the credibility of the program. Participants further stressed the importance of a personalized approach, adjusting recipes to meet their dietary restrictions and preferences	“It was really helpful; it is like having an expert in your pocket. If you put in the effort and make this recipes and you come up with this road block there is someone there that will help you to figure it out. Because I want the recipes, at the time I was trying to avoid white flour, white rice, all sugars, and at the end of the recipe he (C4V Chef) would tell me how to adapt.”
The provision of culinary tips, tricks, and tools to facilitate cooking while experiencing fatigue	Participants in the program were advised to work with or around their fatigue, rather than attempting to re-establish their pre-cancer culinary practices. Participants described a number of energy conservation strategies learned through the program that they found to be of value. Those discussed most frequently included batch cooking and freezing, the use of parchment paper/one pot meals to reduce clean-up, and recipes that used non-perishable or frozen ingredients to limit the need for multiple visits to the grocery store.	“I can make a batch of food and I can divide it into portions and freeze it and then re-heat it afterwards I realized that the amount of work that I put in for let’s say five portions, it pays off. If I was doing individual portions, I would keep doing it and keep doing it, and doing it. I don’t have to waste my time and my energy, so I save [the frozen meals] for days that I don’t have any energy. So that is very useful.” “Pros (of the C4V program) are the tips and tricks and techniques and the kind of flair of making something that is really healthy and nutritious relatively easily, expending a little energy and it’s also fun. You feel supported and have good results. It was overall positive.”
Experiential learning	The opportunity to apply nutritional information and culinary strategies in a hands-on, class-based setting was crucial to participants’ capacity to retain nutritional information as well as practice and refine newly acquired culinary techniques and skills. Access to this kind of experiential learning helped participants to more easily transition these skills from the classroom to the home.	“Being able to do things hands-on, even if only part of the recipe we did ourselves, for me it makes it much more real and more plausible to do it at home. If I just watch, I understand it and see that it is possible but it doesn’t really penetrate, but it is the hands on portion that brings it to life. So I did come home and prepared some food in parchment packages.”
Social support	The group-based environment permitted cancer survivors to interact with as well as learn from each other. This was perceived as valuable for two key reasons. First, it helped to enhance the overall educational experience by fostering group-based question and answer periods. Second, the sharing of experiences between program participants provided opportunities for cancer survivors to normalize and validate their experiences with Cancer-related fatigue (CRF) which can be challenging given the invisibility of this side effect.	“I think connecting with other people who are also going through this struggle; I think connecting with them helps too. It’s not just like a cooking show, when we are meeting together, we kind of share our struggle, even though some of theirs were different than mine. But connecting with them kind of helps you, gives you encouragement. If they are trying, then maybe I should try too.”
**Theme: Areas for program improvement**		
**Sub-themes:**	**Analytic Note**	**Example quote**
One-on-one consultation	Some participants explained that initial one-on-one consultations with each program participant could help to further tailor the content of the class to the unique circumstances and needs of those in the group. This was particularly true for those who felt that their life circumstances or needs were somewhat different from those most typically diagnosed with cancer.	“Because everybody in the class, well, you know (are) older, you have a room of more mature (people). So their metabolism and their goals and expectations are different than mine (as a younger person). So yeah, those one-on-one sessions in the beginning would be nice and helpful I guess, to understand what our expectations and objectives are for attending the session. That and how they can tailor the sessions for us and to help us address our concerns. Or help us get started on our goals.”
A graduated, multi-tiered program	Participants entered the program with different nutritional and culinary backgrounds. It was suggested that taking a multi-tiered approach (e.g., beginner, intermediate, advanced) to delivery would allow participants to enter the program at a level they felt most comfortable with and confident in. This approach was also suggested by those who sought to build upon and advance their knowledge base by graduating to different levels of the program.	P: “I would say it (the C4V Program) is a very positive experience. I was just disappointed that they didn’t have a phase two or phase three. They told me he would run the classes with the same recipes, but I told him that if he was to run the course with different recipes I would definitely go.” I: “In addition to learning new recipes, is there anything else you would hope to get from phase 2 and 3 (of the program)?” P: “Not so much the recipes, but the skills I think, and the experience of actually doing things. The more we do, even though the majority of the stuff is already prepared, the more that we would do ourselves, the more we would start to feel that we are better capable of doing it.”
More in-person cooking sessions	Participants explained that what made this program truly unique was the opportunity to execute recipes in real-time, with the guidance and support of a chef and registered dietitian. Being able to prepare recipes in this context was described as motivating, fostered greater uptake and retention of the recipes and skills, and made meal preparation at home feel more feasible. This was particularly important for those who entered the program with less culinary experience, as they felt additional in-class time was needed to refine and hone their newly-acquired culinary skills.	P: “I think the only (recommendation) is like, we wish there could have been more sessions. More interactive sessions.” I: “Why would that have been beneficial?” P: “… I think that you learn more when you are… you just learn more [in-class] than when you’re given a paper because you get to actually see it, cook it yourself and smell it and taste it. You get to see the finished product. That has a stronger impact and can make it more…it gives me more inspiration.”
A varied approach to the provision of support materials	While some participants enjoyed the convenience of recipe emails and videos, others explained that technology posed a barrier to being able to fully engage with and benefit from the program. It was suggested that a more varied approach to the provision of support materials, designed specifically to the needs and preferences of participants, would help to enhance compliance with the program while at home.	“It would be very nice to have like, a paper copy for example because I don’t have a printer at home. So I have to go back and check the online part. For me, it’s easier to have the paper in front of me because again, we’re talking about fatigue and mental fatigue. What I see is that if I have to go to the computer, turn it on, and look for the recipe, my energy is low there is not motivation. Having my paper recipe in front of me is easier.”

**Table 4:** Estimated means from linear mixed models.

Covariate	Time Point	N	Mean (SE)	95% CI	Difference in the Estimates(SE)	*p*-Value
**Fatigue:** **FACT-F Total Score**	T0	56	23.45 (1.26)	20.94–25.97	-	-
	T1	46	28.37 (1.34)	25.71–31.05	4.91 (1.19)	<0.0001
	T2	42	31.21 (1.38)	28.47–33.95	7.75 (1.24)	<0.0001
**Disability:** **WHODAS 2.0 Total Score**	T0	56	16.12 (0.96)	14.22–18.02	-	-
	T1	44	14.57 (1.02)	12.54–16.61	−1.55 (0.85)	0.072
	T2	42	13.69 (1.03)	11.63–15.74	−2.43 (0.86)	0.006
**Energy:** **Profile of Mood State (POMS)-Vigor Total Score**	T0	57	9.41 (0.61)	8.21–10.62	-	-
	T1	46	10.87 (0.65)	9.59–12.16	1.46 (0.60)	0.018
	T2	41	11.02 (0.67)	9.68–12.37	1.61 (0.64)	0.013
**Confidence in Managing Fatigue** **Total Score**	T0	56	23.37 (0.93)	21.52–25.23	-	-
	T1	45	31.11 (1.09)	28.91–33.31	7.74 (1.28)	<0.0001
	T2	39	34.44 (0.96)	32.51–36.36	11.07 (1.30)	<0.0001

CI: Confidence interval; SE: Standard Error. Participants completed the baseline assessment (T0).Participants completed the post-intervention assessment (T1). Participants completed the three-month post-intervention assessment (T2).

**Table 5:** Qualitative table for clinical outcomes.

Theme: Impact of the C4V Program		
Sub-Themes:	Analytic Note	Example Quote
Improved motivation	Many described losing their motivation to cook because of the limitations posed by fatigue. The thought of cooking as they once had became overwhelming, with many participants opting for fast food instead. Recipes and culinary strategies provided through the C4V program made meal preparation feel more attainable, enhancing feelings of motivation.	“When you finish the program, you are so eager that you come home and prepare the dish the next day. Yes, you get more motivated, I found that I was more motivated after… It also got me excited and interested to make the dishes. And I like cooking and trying new things, so it got me motivated and excited to try the new recipes.”
Improved self-efficacy	Motivation paired with culinary strategies that considered the limitations of fatigue helped many participants to push through their fatigue to make “healthier choices” that they felt more confident with. Participants described feeling better able to apply the skills they learned to implement dietary behavior changes within their daily lives, employing energy conservation techniques to cook while experiencing fatigue.	“(The C4V program was) the game changer. I just picked up so many helpful tips that changed the way my husband and I are now eating. We’re making healthier choices, better choices. It just gave me that boost that I needed to get over the hump of the extraordinary fatigue that happens as a result of chemotherapy.” “There was definitely one day that I clearly remember thinking that I am so tired, and there is no way I’m eating anything healthy. (But then) I thought that I could just buy the fish and cook it. (…) Normally to shop and cook in the same day even if I wasn’t tired would seem impossible (…) So C4V is empowering and skill building in terms of dealing with the fatigue.”
Improved control	Many participants explained that they emerged from the C4V program with important knowledge and skills that enhanced their capacity to eat well. Establishing a sense of control over ones diet was one way participants began to return to normal and gain an improved sense of control over their lives.	“Instead of just being depressed that I don’t know how to feed myself and eating something from take out, now even if I’m tired, I know that I can stop at a grocery store, even stopping at a grocery store would be impossible, I stop at a grocery store and cook it on the same day. Like fish packs, I can go to the grocery store, come home, and within 20 min have something healthy to eat. Even when I am really tired, it still seems like a feasible idea. What this means is a possibility to eat better.”
Improvements to overall fatigue	A few participants explained that the nutritional knowledge and culinary strategies learned through C4V helped them to work around their fatigue to cook more often and felt that they were eating healthier as a result, which directly impacted their fatigue. Others felt that this helped to enhance their energy levels, facilitating engagement in other activities (e.g., physical activity) that were believed to collectively promote reduced fatigue.	“I’ll tell you up front, I think that my energy levels turned around as a result of the (C4V) program... I was eating better, cause I was shown some of the shortcuts, my body was absorbing nutrients that it hadn’t had before, and so I had more energy to go for a walk. I had more energy to prepare a nutritious meal. To me this program was just so significant it should be required (both laugh) for everybody in their cancer treatment. I mean what’s more important than what we put in our bodies?” “Managing fatigue is really about, for me, planning your day… (because of the C4V program) I have new preparation skills that help me to plan ahead, or have things ready frozen and ready to eat. I think that definitely… there are days where I am too tired to cook and (now) I have things available that aren’t a peanut butter sandwich.” “The granola has become my snack instead of the stuff that I am not supposed to eat. And I have more nutrition for my breakfast. So I think that if I have a more nutritious meal, that helps me to fight my fatigue and my tiredness as well.”
